# Herpes Virus Amplicon Vectors

**DOI:** 10.3390/v1030594

**Published:** 2009-10-29

**Authors:** Suresh de Silva, William J. Bowers

**Affiliations:** 1 Department of Biochemistry and Biophysics, University of Rochester, Rochester, NY 14642, USA; E-Mails: suresh_desilva@urmc.rochester.edu; 2 Center for Neural Development and Disease, University of Rochester, Rochester, NY 14642, USA; 3 School of Medicine and Dentistry, University of Rochester, Rochester, NY 14642, USA; 4 Department of Neurology, University of Rochester, Rochester, NY 14642, USA; 5 Department of Microbiology and Immunology, University of Rochester, Rochester, NY 14642, USA

**Keywords:** herpes simplex virus, HSV amplicon, gene transfer, immunotherapy, integration

## Abstract

Since its emergence onto the gene therapy scene nearly 25 years ago, the replication-defective Herpes Simplex Virus Type-1 (HSV-1) amplicon has gained significance as a versatile gene transfer platform due to its extensive transgene capacity, widespread cellular tropism, minimal immunogenicity, and its amenability to genetic manipulation. Herein, we detail the recent advances made with respect to the design of the HSV amplicon, its numerous *in vitro* and *in vivo* applications, and the current impediments this virus-based gene transfer platform faces as it navigates a challenging path towards future clinical testing.

## 1. Introduction

### 1.1. Overview of Herpes Simplex Virus

Herpes simplex virus Type-1 (HSV-1) is one of the numerous DNA viruses that belong to the *Herpesviridae* family. Some of the other well-known members include: Herpes simplex virus type 2 (HSV-2), human cytomegalovirus (HCMV), Epstein-Barr virus (EBV, also called human herpesvirus-4), Varicella zoster (HHV-3), Kaposi’s sarcoma-associated herpesvirus (KSHV). Among these family members, HSV-1 [1], HCMV [2] and EBV [3] have been mainly utilized for the development of gene transfer vectors. Due to its ability to infect numerous cell types, which includes efficient neuronal tropism, coupled with the comprehensive knowledge that has been garnered with respect to its genome organization and biology, HSV-1 has received much interest for the development of various gene transfer platforms as highlighted in the ensuing sections.

#### 1.1.1. HSV-1 Structure

**Figure 1.**
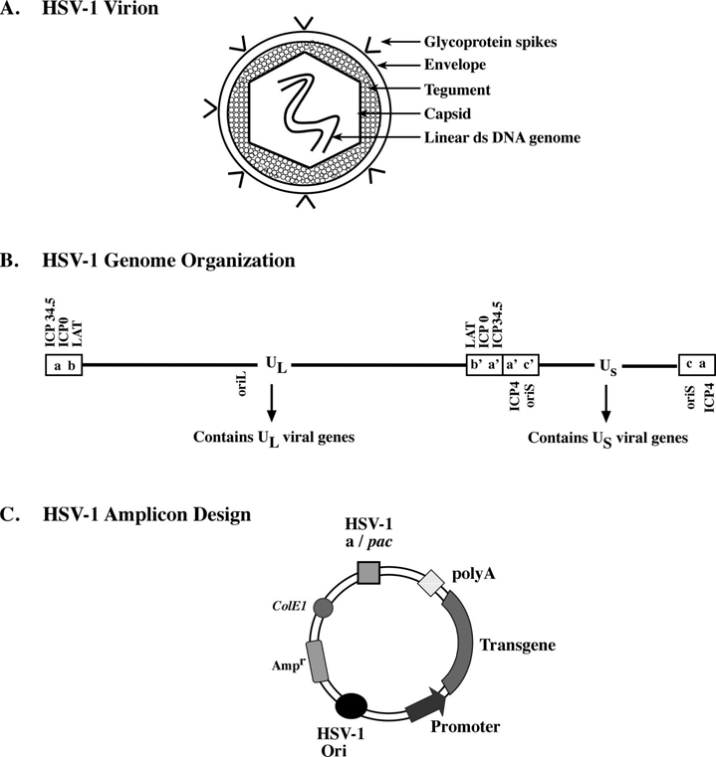
Schematic representation of the HSV-1 virion, its genomic organization, and the basic design of the HSV-1 derived amplicon plasmid. **(A)** The mature wild-type HSV-1 virion is composed of 4 sub-compartments: envelope, tegument, capsid, and the 150-kb linear double-stranded DNA genome. The envelope contains glycoprotein molecules involved in the cellular binding and viral entry processes of HSV-1 infection. (**B)** The linear double-stranded DNA genome of wild-type HSV-1 encodes approximately 80–85 viral genes, which are located in unique long (U_L_) and unique short (U_S_) regions within the genome. Inverted repeat elements (ab, b’a’, a’c’, and ca) flank the unique regions and contain packaging signals required for cleavage and packaging of the replicated viral genome into virions and isomerization of the U_L_ and U_S_ genomic segments. Three viral origins of replication are located within the genome with 2 in the U_S_ region (oriS) and 1 in the U_L_ region (oriL). Several viral genes whose functions are discussed are demarcated. (**C)** The HSV-1 derived amplicon plasmid contains a single oriS or oriL and an “a” site and is devoid of all viral genes. A bacterial origin of replication (ColE1) and an antibiotic resistant gene (Amp^r^) is included for bacterial propagation of the plasmid. A transgene unit-of-interest can be cloned into the HSV-1 amplicon using standard molecular cloning techniques and packaged into HSV-1 amplicon viral particles using helper virus-based or helper virus-free packaging methodologies. (Panel **B** was adapted from [17]).

Members of the herpesviridae family share common structural features exemplified by the prototype, HSV-1. The mature HSV-1 virion is composed of four spatially distinct sub-compartments (Figure 1A).

(1) The core of the virion contains a 152-kb double-stranded linear DNA genome [4], which encodes ∼80–85 viral genes that are arranged as unique long (U_L_) and unique short (U_S_) segments (Figure 1B). These regions in-turn are flanked by inverted repeat sequences (designated ab, b’a’, ac, c’a’) [5], and contain sequences required for cleavage/packaging of the HSV-1 genome, termed “*a*” sites [6]. Additionally, the HSV-1 genome harbors three lytic origins of replication, with two located within the unique short (*oriS*) segment and one in the unique long segment (*oriL*) [7]. Approximately half of the viral genes have been shown to be dispensable for replication of the virus in cultured cells, and thus can be replaced by exogenous genetic material, which has been the premise for the development of HSV-1-based vectors for gene therapy [8].

(2) The HSV-1 genome is enclosed within an icosahedral capsid, which consists of 162 capsomers made up of four capsid proteins: VP5, VP26, VP23, and VP19C [9]. Furthermore, encapsidation and release of viral DNA occurs through a portal located within the capsid, which is formed by a dodecamer of the pUL6 protein [10,11].

(3) Surrounding the capsid is a proteinaceous layer referred to as the tegument [12]. The tegument contains primarily virus-encoded proteins involved in transcriptional regulation of immediate early (IE) viral genes (e.g. VP16), and regulation of host-cell transcription (virion-host-shutoff protein). In addition, the tegument also contains the VP22 protein, which has been implicated in the stabilization of certain viral proteins such as gE, gD and ICP0 [13], and involved in viral spread during lytic infection. However, a recent study by Loret and co-workers demonstrated that specific host cell proteins are also present in modest amounts in the HSV-1 tegument [14].

(4) Finally, a trilaminar lipid membrane, termed the envelope, surrounds the tegument protein layer. Embedded within the envelope are 10 viral glycoproteins that facilitate receptor-mediated cellular entry during viral infection. Among these, glycoprotein B (gB), gC, gD, gH, and gL are important for cellular attachment, fusion, and internalization of the virus [15,16].

#### 1.1.2. Virus entry and delivery of genetic payload

The cascade of events that transpire following HSV-1 attachment to the target cell and subsequent delivery of its genetic payload to the nucleus have been meticulously studied. Initially, the virion docks with the surface of the target cell membrane via the interactions of the heparan sulfate binding domains in viral glycoprotein B (gB) [18] and gC [19] with target cell glycosoaminoglycan receptors, which include the Herpesvirus entry protein A (HveA) [20] and 3-O-sulfated heparan sulfate [21], respectively. In addition, a recent study by Satoh *et al.*, revealed a novel binding interaction between gB and the paired-immunoglobulin-like type 2 receptor-alpha (PILRα) [22], which resulted in virus entry and cell fusion dependent on the binding of gD to its cellular receptor, nectin-1 (previously known as HveC) [23]. Furthermore, HveB (nectin-2) [24] and nectin-3 [25] have been implicated as cellular receptors for herpes virus entry. Following “adsorption” of HSV-1 to the cell surface, gD binds to nectin-1, which induces a conformational change in gD enabling it to interact with gB and/or the heterodimer gH-gL [26]. These events result in the fusion of the viral envelope with the cell membrane, whereupon the capsid is released into the cytoplasm. At this point the tegument proteins are shed into the cytoplasm and the capsid migrates towards the nucleus along the intricate microtubule network of the host cell [27]. The HSV-1 genome is translocated subsequently through the capsid portal into the nucleus via the nuclear pore complex [28]. Once inside the nucleus the viral genome circularizes in the presence of infected-cell protein 0 (ICP0) [29,30] and remains in an episomal form [31], where it can either undergo replication or establish latency.

#### 1.1.3. Lytic *vs.* latent infection

Once its genome is situated within the host cell nucleus, wild-type HSV-1 can initiate a lytic phase, which leads to productive infection or establish latency in the nuclei of sensory neurons upon retrograde transport of the virus. The molecular and viral determinants that dictate which phase the virus will enter are still under investigation. During lytic infection viral genes are expressed in a tightly regulated manner initiating with the expression of five immediate-early (IE) genes (ICP0, ICP4, ICP22, ICP27, and ICP47) via the trans-activating role of the viral tegument protein, VP16 [32]. Next, the IE gene products ICP4 and ICP27 induce the expression of the early (E) genes required for the replication of viral DNA [33,34]. Finally, the late (L) genes are expressed, which mainly encode structural proteins involved in virion assembly. This precisely timed cascade of viral gene expression results in the production of infectious HSV-1 virus particles, which can transport anterogradely to the termini of the axon where they fuse with the cell membrane and are released into the extracellular space. Conversely, the HSV-1 genome can establish latency at which point the only genes that are transcribed are the viral latency-associated transcripts (LATs), whose functions have yet to be fully elucidated (reviewed in [35]). Recent studies have demonstrated that these non-protein coding LATs play a role in regulating the assembly of facultative heterochromatin on lytic gene promoters, thereby inducing transcriptional repression [36,37]. Moreover, Gupta and colleagues have identified and characterized a microRNA (miR-LAT) generated from the exon 1 region of the HSV-1 LAT gene that confers resistance to apoptosis by modulating transforming growth factor (TGF)-beta signaling [38]. Alterations in virus-host interactions can lead to the “reactivation” of the latent HSV-1 genome resulting in a productive infection.

### 1.2. HSV-1 vector generation

The HSV-1 genome contains a significant portion of viral genes that are considered “non-essential” and can be deleted without affecting viral replication in cultured cells. These findings have paved the way for the generation of a number of HSV-1-derived vectors: conditionally replicating vectors, replication-defective vectors, and amplicon-based vectors (reviewed in [39]). Conditionally replicating HSV-1 vectors are capable of replicating only in certain cell types and tissue types *in vivo* due to the deletion of non-essential viral genes (e.g. thymidine kinase and ICP34.5) [40]. Such vectors have been typically used in the development of therapies for malignant brain tumors (e.g. glioblastoma multiforme, GBM), and are referred to as oncolytic HSV-1 vectors. Since replication of these vectors is restricted to rapidly dividing cancer cells, it has been possible to employ suicide gene therapy for the targeted destruction of malignant cells [41]. On the other hand, replication-defective recombinant HSV-1 vectors have been deleted in the viral genes essential for lytic replication and reactivation (*i.e.* the immediate early genes ICP0, ICP4, ICP27, and ICP47), but retain the ability to establish latency [42,43]. Consequently, the generation of replication-defective recombinant HSV-1 particles necessitates the use of complementing cell lines for the production of the deleted viral gene product, which is essential for replication and virion production. The ability for these vectors to maintain latency within the transduced cell has enabled the design of gene transfer modalities using replication-defective HSV-1 vectors for the treatment of certain neurodegenerative diseases and chronic pain (reviewed in [17]). Others have reported on the use of disabled infectious single cycle-herpes simplex virus (DISC-HSV) for anti-tumor applications. These gH-deleted and cytokine-expressing HSV-1 vectors efficiently transduce various tumor cell lines and offer a means to develop efficacious cell-based vaccines (reviewed by [44]). Spaete and Frenkel reported the generation of another type of replication-defective HSV-1 vector via the incorporation of a single origin of replication (ori_S_) and a single packaging/cleavage signal (pac) from the wild-type HSV-1 genome into a standard bacterial plasmid, which they termed “amplicon” [1] (Figure 1C). A transgene expression cassette can be cloned into the amplicon plasmid, and subsequently replicated and packaged into defective viral particles using a number of strategies. The versatility of this category of HSV-1 vectors will be further detailed below.

## 2. HSV-1 Amplicon Vectors

### 2.1. Types of HSV-1 amplicon vectors

**Figure 2.**
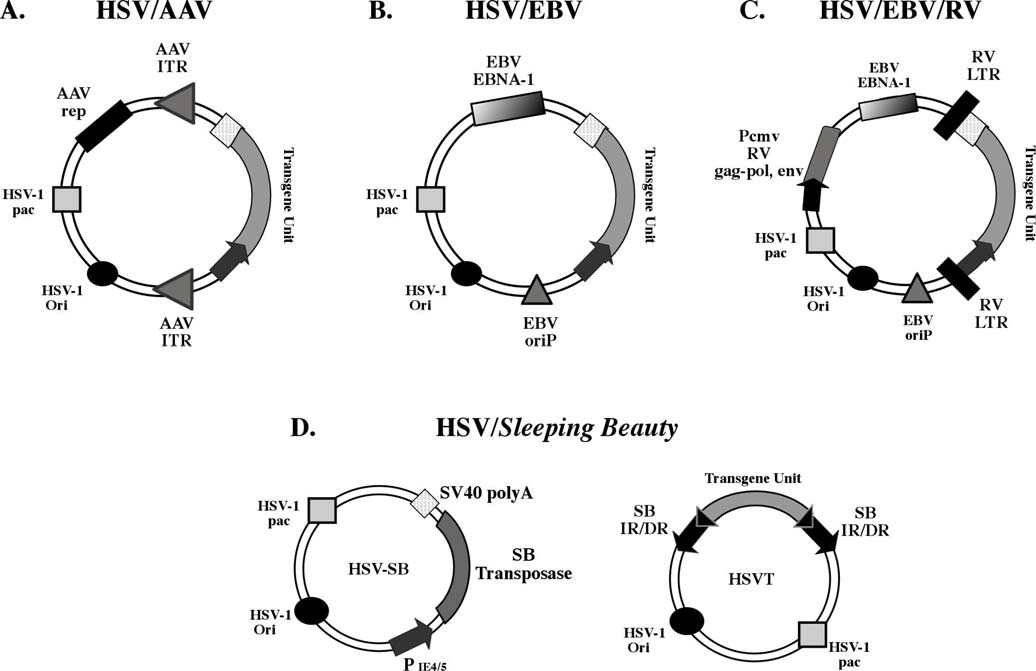
Schematic representation of currently available hybrid HSV-1 amplicon vector platforms. **(A)** The HSV/AAV hybrid amplicon contains the inverted terminal repeats (ITRs) of the adeno-associated virus (AAV), which have been designed to flank the transgene unit-of-interest. The AAV Rep open-reading frame is placed outside the ITR-flanked trangene unit and expression of Rep within the transduced cell mediates the integration of the ITR-flanked transgene into the host cell genome, preferentially at the AAVS1 locus in hChr19. (**B)** The HSV/EBV hybrid amplicon contains the Epstein-Barr virus (EBV) origin of replication (oriP) and EBNA-1 gene, which facilitate replication of the HSV-1 amplicon within the transduced cell. (**C)** Components of the Moloney-murine leukemia virus (MMLV) have been used to generate the “tribrid” HSV/EBV/RV vector. The gag-pol, and env genes are located outside the retroviral long terminal repeat (RV-LTR)-flanked trangene unit and are expressed via a cytomegalovirus promoter, which facilitates retroviral vector production in a permissive cell line. The same EBV components are included (as described in **B**) for episomal retention of the amplicon genome. (**D)** The HSV-1/*Sleeping Beauty* (SB) amplicon vector system requires the co-transduction of 2 amplicon vectors for stable chromosomal integration of a SB inverted/direct repeat (IR/DR)-flanked transgene unit catalyzed by the SB transposase enzyme. Panels A-C were adapted from [45].

The basic design of the HSV-1 amplicon has remained unchanged since its inception, but apparent limitations in its application have prompted the development of hybrid amplicon vector platforms via the incorporation of genetic elements from other viruses as well as from non-viral vector systems (Figure 2). This has been facilitated by the amenability of the HSV-1 amplicon to genetic manipulation, and has expanded its utility as a gene therapy vector.

#### 2.1.1. Conventional amplicons

The conventional HSV-1 amplicon consists of (1) a plasmid backbone harboring a bacterial origin of DNA replication (e.g. ColE1) and an antibiotic resistance gene (e.g., Amp^r^) for propagation in bacteria; (2) two non-coding sequences from the wild-type HSV-1 genome required for replication (ori) and packaging (pac) of the amplicon into infectious particles; and (3) a transgene expression cassette(s). Once packaged into viral particles in the presence of helper functions, the amplicon retains the ability to infect numerous cell types, and its genome maintains an episomal state within the nucleus of the transduced cell. Due to the absence of viral gene expression, the amplicon is completely replication-defective, and its episomal existence results in stable maintenance in post-mitotic cells, but leads to unequal segregation in mitotically active cells. As a gene delivery vector, the conventional amplicon is capable of delivering transgene unit(s) up to 150 kb in size and provides transient gene expression in dividing cells and relatively extended gene expression in post-mitotic cells. Furthermore, since it does not integrate into the host cell genome, the conventional amplicon does not lead to insertional mutagenesis, thus increasing its safety profile as a gene therapy vector.

#### 2.1.2. Episomal amplicons

One perceived limitation of the HSV-1 amplicon is its inability to replicate within the transduced cell, which leads to segregation and loss of transgene expression in dividing cells. Several modifications have been made to the HSV-1 amplicon to either confer replication-competency or incorporate DNA elements that enhance its episomal retention within the transduced cell nucleus (reviewed in [46]). Wang and co-workers incorporated elements from another Herpesviridae family member, the Epstein-Barr virus (EBV), to generate a hybrid amplicon capable of replicating concomitantly with chromosomes in dividing cells to facilitate stable episomal maintenance [47] (Figure 2B). This design involved the EBV latent replication origin sequence (oriP) and the EBV nuclear antigen-expressing gene, EBNA-1, which is the only virus-encoded transactivator of oriP. Based on this hybrid amplicon vector platform, entire genomic loci (e.g. hypoxanthine phophoribosyltransferase (HPRT) [48], and human low-density lipoprotein receptor (LDLR) [49]) have been cloned into “retro-fitted” BAC’s harboring components of the EBV/HSV amplicon system and efficiently packaged into HSV-1 viral particles. Infection of HPRT- and LDLR-deficient cell lines by BAC-based EBV/HSV vectors expressing the HPRT and LDLR genes, respectively, resulted in episomal retention and prolonged transgene expression.

To circumvent the use of viral components such as EBNA-1, which is immunogenic and may exhibit oncogenic properties, Lufino and colleagues recently utilized a human episomal retention element (scaffold/matrix attachment region (S/MAR)) from the human β-interferon gene to generate a novel HSV-1 amplicon-based episomal vector, designated iBAC-S/MAR [50]. The S/MAR element is known to be involved in chromatin function, DNA replication and gene expression via its interaction with the nuclear matrix of the cell. Using the iBAC-S/MAR vector, Lufino and colleagues delivered the LDLR gene containing the genomic locus into CHO *ldlr*^−/−^ a7 cells and demonstrated 30% efficiency in episomal maintenance of the vector and physiological levels of LDLR gene expression up to 70 cell generations under antibiotic selection and up to 100 cell generations in the absence of selection.

In an alternate approach, Moralli and co-workers generated a novel HSV-1 amplicon vector based on a human artificial chromosome (HAC), which allows for segregation and retention of the amplicon vector during cell division [51]. The HAC used in this study contained 17 alphoid DNA (α-satellite higher-order repeat sequences) and the entire HPRT gene locus and was adapted to the HSV-1 amplicon for packaging into infectious particles. The HPRT-expressing HAC-based HSV-1 vector was capable of efficiently transducing HPRT-deficient fibrosarcoma cells and demonstrated episomal retention and expression of HPRT up to 3 months. These reports reveal the potential of using various viral and human-derived genetic elements to increase episomal maintenance of the HSV-1 amplicon and extend transgene expression, but further improvements and *in vivo* testing are required to test the true potential of each of these iterations for therapeutic applications.

#### 2.1.3. Integration-competent amplicons

One of the main features of the HSV-1 amplicon is the episomal existence of its genome within the nucleus of the transduced cell. It has been long believed that stable integration of the transgene unit within the transduced cell could provide the means to extend expression duration and retention of the transgene. This concept has led to the development of hybrid amplicon vector platforms engineered to contain elements from viral and non-viral systems that facilitate the integration of the amplicon-ferried transgene unit into host cell chromosomes (reviewed in [52]). The first hybrid amplicon vector to be generated was the HSV-1/adeno-associated virus (AAV) hybrid amplicon, which incorporated the inverted terminal repeat (ITR) elements and the Rep gene from AAV into the HSV-1 amplicon backbone ([53] and reviewed in [54]). These elements are directly involved in the integration of the AAV genome into the AAVS1 site located on human chromosome 19q13.3-qter during the latent phase of wild-type AAV infection. In the basic HSV/AAV amplicon design, the transgene expression cassette is flanked by the ITR elements within the HSV-1 amplicon, while the Rep gene is placed outside of the ITRs to preclude its integration (Figure 2A). Cell culture-based testing and *in vivo* testing has established that the HSV/AAV amplicon platform is competent in stably integrating the ITR-flanked transgene cassette within the genome resulting in sustained transgene expression [55,56]. Furthermore, analysis of stably transduced clonal lines has revealed that the ITR-flanked transgene cassette integrates at a significant efficiency into the AAVS1 site located on hChr19. In a recent study, Oehmig and co-workers demonstrated that a BAC-based HSV/AAV amplicon platform specifically integrated the entire human β-galactosidase (β-GAL) gene locus (133-kb) into the AAVS1 locus in 33% of the analyzed glioblastoma (Gli36) clones transduced with the vector [57]. Furthermore, HSV/AAV-mediated integration of the β-GAL gene resulted in elevated enzyme activity (>2-fold) in approximately 80% of Gli36 clones determined at 15–18 weeks post-infection.

One of the noted drawbacks of the HSV/AAV amplicon design has been the cytotoxic and inhibitory effect of Rep elicited during HSV/AAV amplicon production, which has made it challenging to produce high-titer amplicon vector stocks [53,58]. Thus, modifications have been introduced into the HSV/AAV platform to mitigate the inhibitory effects of Rep during amplicon packaging. One such strategy reported by Liu and colleagues involved the design of a *loxP*-flanked ITR-cassette containing the Rep68/78 open-reading frame strategically placed within the HSV-1 amplicon [59]. This design ensured that Rep would be expressed only in the presence of Cre recombinase via the p5 promoter element, Thus, Rep would not be expressed during packaging of the HSV/AAV amplicon, but would facilitate AAVS1 integration of the ITR-flanked transgene in a Cre-expressing cell line. Their results revealed that this novel design increased amplicon titers ten-fold higher than the original HSV/AAV amplicon design, and also demonstrated that integration of the ITR-flanked transgene unit at the AAVS1 site occurred in 70% of the stably transduced Cre-expressing 293^+^ clones. However, it should be noted that stable integration mediated by HSV/AAV amplicon vectors do not exclusively occur at the AAVS1 site, since studies have shown that integration also occurs at other sites within the transduced cell genome. In aggregate, the HSV/AAV hybrid amplicon is an attractive gene therapy platform with the potential for site-specific integration of large therapeutic transcription units pending further improvements and rigorous *in vivo* testing.

“Tribrid” HSV-1 amplicon vectors have also been generated using components from the HSV-1 amplicon (ori and pac), EBV (oriP and EBNA-1) or AAV (ITRs and Rep), and moloney murine leukemia virus [retrovirus vector sequences, and the retroviral gag-pol, and env genes (GEP)] with the intention of generating a novel vector capable of converting glioma cells into retroviral packaging cells [60,61] (Figure 2C). Since the retrovirus vector sequences and the collection of GEP genes are sufficient for producing retroviral vectors, the HSV/EBV or HSV/AAV vector would serve as the delivery vehicle and its episomal retention or integration into the genome would facilitate continuous production of retroviral vectors from glioma cells. Consequently, *in vivo* and *ex vivo* testing by Hampl and co-workers revealed that the HSV/EBV/RV amplicon was indeed capable of converting both on-site tumor cells as well as tumor cells *ex vivo*, into retroviral producer cells as evidenced by a four-fold increase in transgene expression in the tumor compared to standard amplicon vectors [62]. This study provided proof-of-concept for the use of HSV/EBV/RV vectors for producing and expanding the delivery of retroviral vectors bearing therapeutic genes for the on-site treatment of glioblastomas.

Using the elements from the Tc-1 like *Sleeping Beauty* (SB) transposon system, a bi-partite HSV/SB amplicon vector platform has been designed for stable integration of transgene units to achieve long-term expression [63,64]. This two-vector system consists of an “effector” amplicon, which expresses the SB transposase, and an “integrator” amplicon that contains a reporter transgene unit flanked by the inverted/direct repeat elements (termed IR/DR) of SB (Figure 2D). Upon co-transduction of the two amplicons, the SB transposase catalyzes the precise excision and subsequent integration of the IR/DR-flanked transgene unit into a random TA dinucleotide site within the mouse genome. *In utero* application of the HSV/SB amplicon vector system in the developing mouse brain resulted in the integration of the reporter transgene in the transduced neural precursor cell population leading to brain-wide expression of a *lacZ* reporter transgene detectable up to 90-days post-birth via immunohistochemistry. However, the random integration profile of the SB system raises the safety concern with possible insertional mutagenesis, hence strategies are currently being devised to alter the integration profile of the transposase to achieve site-targeted integration. In aggregate, the versatility of the plasmid-based HSV-1 amplicon design has allowed for extensive genetic manipulation of the vector to generate hybrid vector platforms capable of extended episomal retention, genomic integration of the amplicon-ferried transgenes, and site-specific integration, qualities that significantly expand its utility for efficient *in vivo* gene transfer.

### 2.2. HSV-1 amplicon vector packaging systems

#### 2.2.1. Helper virus-based packaging system

The traditional helper virus-based packaging system involves a replication-defective HSV-1 virus, which is devoid of one or more key viral genes, to provide the functions necessary for packaging the amplicon into infectious viral particles [65]. Initially, the amplicon plasmid is transfected into eukaryotic cells that have been stably transfected with a plasmid expressing the viral gene deleted from the helper virus and the cell monolayer is subsequently super-infected with the helper virus. The most widely used systems employ helper viruses that are deleted of both copies of the HSV-1 IE3 gene, which encodes the ICP4 protein, and stable cell lines that complement the IE3 gene function (*i.e.*, RR1 and E5 cells) [65,66]. Coordinated expression of viral genes from the helper virus results in the replication of the transgene-harbored amplicon plasmid in a mono-directional manner via a rolling circle mechanism [7]. As mentioned previously, this leads to the generation of a concatemer consisting of several copies of the amplicon unit arranged in a head-to-tail configuration, which is subsequently packaged into viral particles as 150-kb units. The number of copies contained within a concatemeric genome is determined by the original size of the transgene-harbored amplicon plasmid. For example, if the size of the amplicon plasmid is 10 kb, the packaged viral particle will contain a concatemeric amplicon genome consisting of 15 copies of the amplicon.

**Figure 3.**
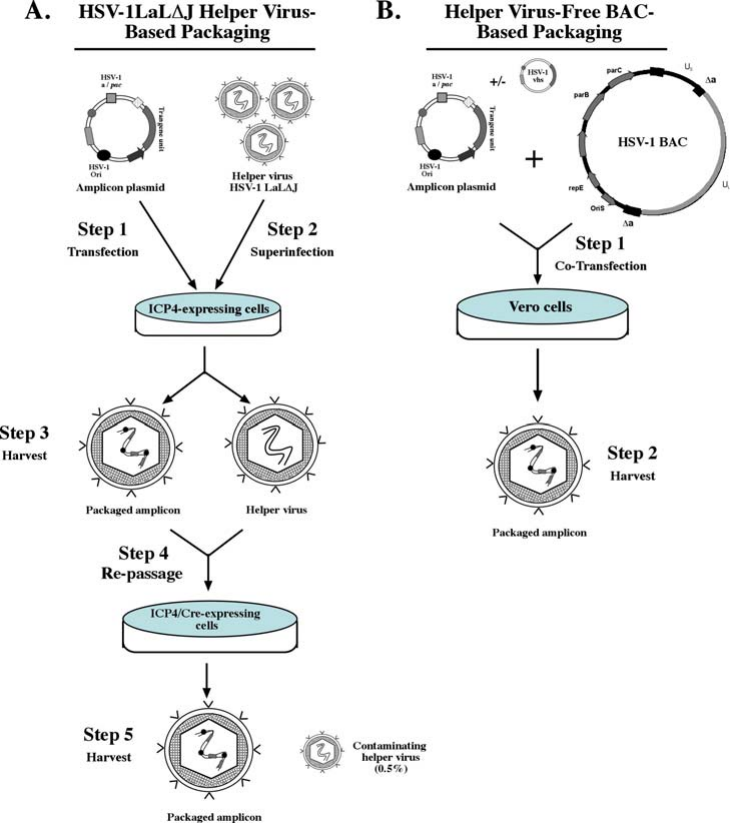
Schematic representation of the latest packaging systems utilized for amplicon vector production. **(A)** The HSV-1LaLΔJ helper virus-based amplicon packaging technology involves initial transfection of the transgene-harbored amplicon plasmid into an ICP4-complementing cell line with subsequent superinfection with the HSV-1LaLΔJ helper virus, which contains a unique ‘floxed’ packaging signal designed to reduce helper virus contamination. The resultant viral stock from the ICP4-complementing cell line that contains both packaged amplicon particles and helper virus particles (at approximately a 1:1 ratio) is used to infect a second cell line, which expresses ICP4 and Cre recombinase. Recombination of the *loxP*-sites via Cre recombinase results in the deletion of the packaging signal from the helper virus, which renders its own genome packaging-defective and thereby reduces the titers of helper virus present (0.5%) in the final harvested amplicon vector stock. (**B)** The helper virus-free packaging technology involves the co-transfection of a permissive cell line such as Vero 2-2 cells with the transgene-harbored amplicon plasmid and a bacterial artificial chromosome (BAC) designed to harbor the HSV-1 genome deleted in the packaging signals. An accessory plasmid expressing other HSV-1 proteins, such as vhs, can be included to enhance titers. The HSV-1-BACΔpac genome has been strategically designed to preclude its packaging into viral particles, thereby eliminating the presence of helper virus contamination in the final amplicon vector stock. (Figure was adapted from [45,68]).

Helper virus-based packaging systems pose a problem in that resultant HSV-1 amplicon preparations are contaminated with appreciable numbers of helper virus particles due to their inherent structural similarity. This makes it challenging to generate pure amplicon vector stocks via currently employed purification techniques. High titers of contaminating helper virus have been shown to be cytotoxic to transduced cells, and are potent in inducing transient, but significant, inflammatory responses when administered *in vivo* [67]. Furthermore, it is theoretically possible that recombination between the helper virus and packaging cell genomes could occur, leading to the reversion of the helper virus to wild-type virus, which further increases the risks associated with helper virus-based packaging systems. Refinements have been made to the above process to minimize the levels of contaminating helper virus in amplicon stocks, and accordingly mitigate cytotoxic effects associated with helper virus-based packaging systems (reviewed in [68]). Logvinoff and colleagues ectopically engineered a unique *loxP*-flanked “pac” site within the gC locus of the helper virus, which could be specifically deleted from the helper virus via packaging on a Cre recombinase-expressing cell line (TE-Cre30), thus precluding its ability to package into viral particles [69]. This system was termed HSV-1 LaL, and amplicon packaging using this system resulted in a high-titer amplicon vector stock (Figure 3A). However, it was observed that complete excision of the pac signals in all helper virus genomes was unattainable by the Cre/*loxP* system as evidenced by the presence of replication-competent helper virus in the final amplicon stock. To further improve this system, Zaupa and colleagues subsequently deleted the known neurovirulence factor ICP34.5-encoding gene (γ34.5) and the ICP4-encoding gene IE3 that flank the “floxed” pac site to generate the HSV-1 LaLΔJ helper virus [70]. Production of amplicon viral particles in a two-step process using the HSV-1 LaLΔJ helper virus in cell lines that complement the expression of ICP4 and Cre recombinase resulted in a significant reduction in the level of contaminating replication-incompetent helper virus (down to 0.05%–0.5%), while maintaining amplicon titers at ∼2×10^7^ TU/ml. Recently, Halterman and colleagues deleted the U_L_41 gene, which encodes for the virion host shutoff (vhs) protein, from the helper virus genome and tested its effect during helper virus-based amplicon packaging [71]. Their results revealed that the absence of vhs during helper virus-based packaging resulted in a decrease in amplicon vector titer, but amplicon stocks imparted less cytotoxicity compared to control amplicon stocks generated in the presence of vhs. Furthermore, expression of vhs mutants deficient in mRNase activity during amplicon packaging in the presence of the U_L_41-deleted helper virus was able to improve amplicon titers while minimizing cytotoxicity. In summary, current helper virus-based packaging technologies are capable of producing high-titer amplicon vector stocks with significantly reduced helper virus contamination, but further improvements are required to completely eliminate the presence of helper virus. Accordingly, alternative methods have been developed to completely eliminate the use of helper virus during amplicon vector packaging via the use of helper virus-free systems, which will be discussed in the next section.

#### 2.2.2. Helper virus-free packaging systems

Cunningham and co-workers provided the molecular framework upon which the first alternative strategy to package amplicon vectors was created. This group constructed a set of five overlapping cosmids, which were designed to provide full coverage of the entire HSV-1 genome [72]. Transfection of these overlapping cosmids into eukaryotic cells resulted in the reconstitution of the HSV-1 genome via homologous recombination of the cosmids. Subsequently, Fraefel and co-workers deleted the cleavage/packaging (pac) signals from the cosmids and demonstrated that co-transfection of a reporter amplicon plasmid together with the pac-deleted cosmids resulted in amplicon vector stocks free of detectable helper virus contamination [73]. The complexity of using five HSV-1 cosmids coupled with the relatively low amplicon vector yield (∼10^5^–10^6^ expression units per ml) prompted the development of a simplified, more efficient helper virus-free system, which was accomplished by cloning the entire pac-deleted HSV-1 genome into a single F-plasmid-based bacterial artificial chromosome (termed fHSVΔ*pac*) [74]. Co-transfection of eukaryotic cells with fHSVΔ*pac* and an amplicon plasmid resulted in amplicon stocks up to 10^7^ transducing units per ml (TU/ml) with minimal replication-competent helper virus contamination (shown to be less than 1 replication-competent virus per 1.5×10^5^ TU/ml detected via a standard plaque assay). Subsequently, Saeki and colleagues generated an oversized BAC clone (fHSVΔ*pac*Δ27 0+) via targeted deletion of the gene that encodes ICP27 and inserting ICP0 “stuffer” sequences to increase the size of the original BAC clone to 178-kb [75]. Amplicon vector production in an ICP27-complementing cell line using the fHSVΔ*pac*Δ27 0+ BAC resulted in a further reduction of wild-type revertant virus contamination in amplicon stocks (<1 plaque forming unit per 10^8^ TU amplicon vectors), while generating amplicon titers of 3–10×10^8^ TU/ml after concentration. Other BAC-based helper virus-free amplicon packaging platforms have been created (Figure 3B), which generate moderate-titer stocks with very low frequencies of wild-type revertant contamination [76,77].

Despite these technological advances, amplicon titers achieved via helper virus-based amplicon packaging systems are still higher than those achieved using helper virus-free systems. One reason why helper virus-free systems lag in this respect may be due to preformed viral tegument proteins present in the helper virus that have been shown to prime the cell for virus production via the trans-activation of IE genes (*i.e.*, VP16), and also by regulating cellular and viral transcript levels [*i.e.*, virion host shutoff protein (vhs)]. Our laboratory previously reported the co-transfection of a plasmid harboring the U_L_41 gene, which encodes vhs, as well as pre-loading the packaging cells with a VP16-expressing plasmid construct during BAC-based packaging as a means to enhance amplicon titers [77]. This dual-hit strategy resulted in a 50-fold increase in amplicon vector titers compared to controls, and furthermore significantly reduced the pseudo-transduction phenomenon observed with stocks generated via the first-generation helper virus-free packaging system. However, further optimization and refinement of currently used helper virus-free packaging systems will be required to achieve titers that are high enough to feasibly test the clinical potential of amplicon vectors.

### 2.3. HSV-1 virion engineering for targeted transduction

To the extent that the HSV-1 genome has been manipulated for vector generation, components of the virion envelop involved in host cell binding and virus entry such as gC, gB, and gD have also been the focus of genetic manipulation to alter HSV-1’s natural tropism. As described previously, these glycoprotein molecules are responsible for recognizing ubiquitously expressed cellular receptors (HveA, Nectin-1, 3-O-sulfated heparan sulfate) during viral docking and entry, which renders multiple cell types permissive to HSV-1 infection. However, treatment of certain genetic conditions may necessitate targeted delivery of the therapeutic gene to preclude deleterious side effects, which could be caused by expression of the therapeutic gene in unintended cell types. Thus, attempts have been made to re-target and restrict infection to specific cell types via HSV-1 vectors by genetically replacing the heparan sulfate-binding domain of glycoprotein C (gC) with a ligand capable of binding a specific receptor/protein on a target cell surface without hindering viral entry. A proof-of-concept study was initially performed by replacing the heparan sulfate-binding domain of gC with the erythropoietin hormone (EPO) in a recombinant HSV-1 virus deleted for gC and the heparan sulfate-binding domain of gB (KgBpK^−^gC^−^) [78]. Recombinant HSV-1 virions containing the gC-EPO chimeric proteins in their envelope were capable of binding to EPO receptors and stimulating the growth of an EPO-dependent cell line (FD-EPO). However, binding of the gC-EPO recombinant virus did not result in a productive infection since the FD-EPO cell line is non-permissive to HSV-1 infection. Subsequently, Argnani and colleagues utilized a similar strategy where the heparan sulfate binding domain of gC was replaced by a 27-amino acid active peptide (termed preS1ap) found in the hepatitis B virus entry molecule, PreS1 [79]. Incorporation of this chimeric gC:preS1ap molecule into recombinant virions (KgBpK^−^gC:preS1ap) resulted in the specific binding of the recombinant virus to a human hepatoblastoma cell line (HepG2) leading to a productive infection compared to the parental KgBpK^−^gC^−^ mutant virus. It has also been shown that glycoprotein D (gD) can be replaced by the vesicular stomatitis virus glycoprotein G spike (VSV-G) in the envelope of a pseudotyped recombinant HSV-1 virion and still retain virus entry activity and initiate a productive infection in a gD-complementing VD60 cell line, albeit at a 50% reduced level compared to a wild-type virus containing gD [80]. Since recombinant HSV-1 virions and HSV-1 amplicons are identical in their structural components such strategies can be applied to re-target the HSV-1 amplicon.

Accordingly, Grandi and co-workers were successful in re-targeting an HSV-1 amplicon virus by incorporating a chimeric form of the gC protein containing a His-tag in place of the heparan sulfate binding domain [81]. This was accomplished by expressing the gC-His tag fusion protein from the amplicon plasmid (pCONGAH) during packaging using a gC-deleted helper virus. Assessment of the binding specificity of the His-tagged-gC amplicon virus revealed a four-fold increase in binding to a pseudo-His-tag receptor expressing 293 6H cell line compared to a parental 293 cell line. *In vivo* application of a HSV-1 amplicon re-targeting strategy was reported by Wang *et al.*, and Cao *et al.*, wherein helper virus-free HSVlac amplicon particles expressing the β-galactosidase reporter transgene were generated harboring either chimeric gC-GDNF (glial cell derived neurotrophic factor) or gC-BDNF (brain-derived neurotrophic factor) proteins via replacement of the heparan sulfate binding domain in gC with the neurotrophic factor encoding cDNA [82,83]. Both modified amplicon vectors were capable of supporting a 2.2- to 5-fold increase in targeted gene transfer to nigrostriatal neurons, which express the cognate receptors for GDNF (GFRα-1) and BDNF (TrkB), in the rat brain compared to HSV-1 amplicon particles harboring wild-type gC as assessed by immunohistochemistry 4-days post-injection. However, it was shown that both modified amplicon vectors also infected other cell types lacking the neurotrophic receptors in the rat mid-brain, which could be due to the fact that the heparan sulfate-binding domain of gB was not deleted in these virions, thereby allowing for non-targeted infection. These studies highlight the potential for engineering HSV-1 amplicons for targeted infection via the incorporation of ligands capable of binding receptors present on specific cell types.

### 2.4. Immune responses to HSV-1 amplicons

As described previously, packaged HSV-1 amplicon particles share the structural components of the wild-type (wt) HSV-1 virion with the exception of *de novo* viral gene expression. Thus, both iterations of the HSV-1 virus encounter the host immune system in a similar way when initially introduced *in vivo*, but can differ in their respectively induced immune responses (Reviewed in [84,85]). Studies detailing the manner by which wild-type HSV-1 and HSV-1 amplicon vectors interact with the host’s immune system have provided valuable insights into the development of improved amplicon vector design and refined packaging technologies for safe, efficient and long-term transgene expression.

Wild-type HSV-1 is multi-faceted in its ability to successfully evade the immune system and enter into either a productive lytic phase of infection or establish a latent state in sensory neurons. It is known that upon HSV-1 infection, an innate, type I interferon (IFN-α/β) response is triggered via Toll-like receptor (TLR)-dependent and TLR-independent pathways (reviewed in [86]). However, wild-type HSV-1 is able to dampen the IFN anti-viral response via the action of the immediate-early proteins ICP0 and ICP27. These viral proteins inhibit downstream signaling processes that would normally lead to activation of IFN-stimulated genes and production of pro-inflammatory cytokines [87,88]. Furthermore, wild-type HSV-1 is armed with more viral products that render it elusive to MHC class I cytotoxic T-lymphocyte (CTL) recognition, and thus preclude the activation of the complement cascade (reviewed in [89]). This is accomplished through the activities of the gI, gE, and gB glycoproteins, virion host shutoff (vhs), and ICP47 protein contained within the virus. The envelope-resident glycoproteins are involved in masking the virus from host anti-viral immunoglobulins (IgG’s), which include pre-existing anti-HSV-1 antibodies that are present in nearly 90% of human adults [90]. In a phenomenon termed “antibody bi-polar bridging” [91], the gE-gI complex binds to the Fc domain of the anti-HSV-1 IgG, which in turn is hypothesized to bind to gB, gC, and/or gD via its Fab domain. This results in the inability of the bound anti-HSV-1 IgG molecule to activate the complement cascade, which is required for clearance of the virus. In addition, vhs [92] and ICP47 [93] work in concert to interfere with cytotoxic T-lymphocyte (CTL)-mediated recognition of the infected cell via down regulation of MHC class I expression and inhibition of antigenic peptide presentation, respectively. The packaged HSV-1 amplicon particle contains nearly all of the above-mentioned viral gene products (with the exception of ICP0 and ICP27, which may be present in trace amounts in the tegument following expression in the packaging cell line), and has been shown to induce an IFN response upon transduction. However, based on the packaging system used for amplicon production (*i.e.* helper virus-based *vs.* helper virus-free) additional antigenic components derived from either the packaging cell line or the helper virus (present as a co-purifying contaminant) could elicit an immune/inflammatory response. In one of the earliest assessments, Wood and coworkers demonstrated that a β-galactosidase-expressing HSV-1 amplicon packaged in the presence of a temperature-sensitive, recombinant HSV-1 helper virus (*tsK*) induced a vigorous inflammatory response that persisted up to 31 days following stereotactic delivery of the amplicon into the rat dentate gyrus [94]. They proved that the observed inflammation was a consequence of contaminating helper virus particles in the amplicon vector stock. Since then, helper virus-based packaging technology has been modified to significantly reduce the presence of contaminating helper virus (described in a previous section). Recently, Tsitoura and colleagues transduced primary human fibroblasts of limited passage (HFFF-2) with a HSV-1 amplicon expressing the green fluorescent protein (GFP) packaged via the recently developed HSV-1 LaLΔJ helper virus system and monitored the amplicon-induced innate immune response [95]. Their results revealed that HSV-1 amplicon transduction induced an IFN regulatory factor 3 and 7 (IRF3/7)-dependent antiviral response in the HFFF-2 cell line independent of TLR, which resulted in the enhancement of IFN-stimulated gene transcription. This latter study suggests that HSV-1 amplicons are capable of inducing multiple innate immune responses mediated by TLR-dependent and TLR-independent pathways.

As described previously, the advent of helper virus-free packaging techniques based on the BAC and cosmid systems has significantly reduced the severity of immune/inflammatory responses associated with HSV-1 amplicon stocks. *In vivo* profiling of the inflammatory responses in the central nervous system following stereotactic delivery of a β-galactosidase-expressing amplicon (HSVlac) packaged in the presence of a helper virus or helper virus-free packaging technique has been reported [67]. Helper virus-packaged HSVlac-injected C57/BL6 mice revealed elevated transcript levels of pro-inflammatory cytokines (IL-1β, TNF-α, IFNγ), chemokines (MCP-1, IP-10), and an adhesion molecule (ICAM-1) and immune cell infiltration at day 5 post-injection detected by quantitative RT-PCR analysis and immunocytochemistry. Conversely, the helper virus-free HSVlac-injected group, as well as the saline-injected control group, exhibited a transient immune cell infiltration and elevation of pro-inflammatory transcripts due to stereotactic surgery-associated opening of the blood-brain barrier, which resolved within a 3-day period.

The induction of an innate immune response within the host not only affects efficiency of transgene delivery via the HSV-1 amplicon to the target cell, but has also been shown to negatively influence transgene expression. Previous studies have shown that amplicon-mediated transgene expression in the brain, as well as cell types (dividing and non-dividing) in other organs, is transient due to host cell-induced silencing of the transgene-harbored amplicon genome. One of the mechanisms by which transgene silencing occurs was suggested by Suzuki *et al.*, who demonstrated that bacterial sequences contained within a conventional HSV-1 amplicon expressing a firefly luciferase transgene triggered the association of an inactive form of chromatin at high levels within the prokaryotic sequences and subsequently surrounding vector genome regions, following transduction of non-dividing human fibroblasts (MRC9) [96]. This chromatin condensation resulted in rapid transcriptional repression of the luciferase transgene 6 days post-infection. Conversely, a transduced mini-circle (MC) form of an amplicon genome devoid of bacterial sequences showed comparatively lower levels of the inactive form of chromatin correlating with a slower decline in luciferase transcript levels over the 6-day assessment period. They proposed that the bacterial sequences trigger a host immune response capable of silencing the prokaryotic sequences, including the vector-encoded transgene, via heterochromatin deposition.

In an effort to dissect the interplay between the early host immune response following HSV-1 amplicon infection and its effect on transgene expression, Suzuki and colleagues systemically delivered a firefly luciferase-expressing HSV/EBV amplicon (termed pREHZCag-Luc) and discovered that a type-I interferon response (IFN-α/β) followed by transient activation of signal transducers and activators of transcription 1 (STAT1) was rapidly induced in a vector dose-dependent manner in the liver within 12 hours following amplicon injection compared to the saline-treated group [97]. Furthermore, systemic delivery of the luciferase-expressing amplicon in STAT1 knockout mice resulted in luciferase gene expression lasting up to 80-days, limited IFN response, and absence of infiltrating CD11b-positive immune cells in the liver compared to the amplicon-injected wild-type control group. Their investigation into delineating the extended luciferase gene expression in STAT1-knockout mice compared to wild-type mice (*i.e.*, 80-days *vs.* 10-days post-infection) revealed that the type I IFN/STAT1 signaling pathway was responsible for transcriptional silencing of the luciferase transgene-harbored amplicon. Another mechanism by which an “open” chromatin status of the HSV-1 amplicon could be maintained and thus extend transgene expression is via the transient expression of the immediate-early protein ICP0, which has been shown to decrease the association of histone deacetylase 1 (HDAC1) with amplicon DNA in the liver following systemic delivery of an ICP0-expressing HSV-1 amplicon vector (pHGCagK-Lac-ICP0) [98]. Surprisingly, HSV-1 amplicon-mediated expression of ICP0 in the liver did not block the induction of type I IFN response, which has been shown to occur during wild-type HSV-1 infection [99].

In a separate study, Suzuki and co-workers sterotactically delivered the same luciferase-expressing HSV-1 amplicon (pREHZCag-Luc) into the striatum of C57BL/6 mice and demonstrated luciferase expression lasting up to 1-year post-infection, Characterization of the immune response following amplicon delivery in the brain revealed a vector dose-dependent increase in transcript levels of type I IFNs [100]. Further analysis revealed a bias toward a Th1 immune response rather than a Th2 response in the brain. Moreover, their results also revealed that increased transcript levels of immunosuppressive signals, transforming growth factor-β (TGF-β), and IL-10 together with an increased presence of T-regulatory cells (T_reg_) may have been beneficial in maintaining long-term luciferase gene expression in the brain. Based on these studies, it is evident that helper virus-free amplicons elicit a modest but varied immune response depending on the target organ and route of vector delivery. Such host responses influence transduction efficiency and expression duration of the delivered transgene.

### 2.5. HSV-1 amplicons for gene therapy

The ability to deliver exogenous genetic material up to 150 kb, coupled with its capacity to efficiently transduce numerous cell types, including those of the central nervous system (CNS), has positioned the HSV-1 amplicon as a versatile gene transfer vector for the treatment of several disorders and conditions affecting the CNS. The preclinical studies reported previously are summarized in Table 1 and are discussed in further detail below.

#### 2.5.1. Parkinson’s disease

Parkinson’s disease (PD) is a progressive neurodegenerative disorder caused by the selective loss of nigrostriatal neurons, the cells responsible for producing dopamine [101]. Loss of this essential neurotransmitter in the brain results in resting tremor, rigidity, and severe motor function impairment. The most efficacious therapy currently available to alleviate the symptoms associated with PD include administration of L-DOPA (levodopa), which is the precursor to dopamine and is converted to dopamine by the endogenous aromatic amino acid decarboxylase (AADC) present in the surviving dopaminergic neurons [102]. However, since L-DOPA therapy loses its efficacy over time, gene-based therapeutic strategies using viral vectors, including those based upon HSV-1 amplicons, are being created to restore function to the damaged nigrostriatal pathway. Strategies include the restoration of dopamine levels via replacement of genes involved in the dopamine biosynthetic pathway (e.g. tyrosine hydroxylase (TH) and AADC), as well as delivering neurotrophic genes (e.g. glial cell-derived neurotrophic factor (GDNF), and brain-derived neurotrophic factor (BDNF) to protect remaining dopaminergic neurons from further demise.

In a recent study, Sun and co-workers exploited the large transgene capacity of the HSV-1 amplicon and generated a single construct harboring four genes involved in dopamine biosynthesis and transport (TH, GTP cyclohydrolase I, AADC, and vesicular monoamine transporter-2) under the transcriptional regulation of a modified neurofilament gene promoter for long-term, γ-aminobutyric acid (GABA)-ergic neuron-specific gene expression [103]. Subsequent helper virus-free packaging and delivery of this 4-gene-vector into the striatum of 6-hydroxydopamine (6-OHDA)-lesioned rats resulted in the production of dopamine and dihydroxyphenylacetic acid (DOPAC), K^+^-dependent release of dopamine, and high levels of correction of apomorphine-induced rotational behavior observed up to 6 months. In an alternate approach, Sun and co-workers compared the efficacy of neurotrophic factors in protecting nigrostriatal neurons and improve behavioral deficits in a 6-OHDA--lesioned rat model of PD. HSV-1 amplicons were generated to express either GDNF, BDNF, or both under a modified neurofilament gene promoter, packaged via a helper virus-free system, and delivered unilaterally into the striatum of rats followed by intrastriatal 6-OHDA injections [103]. Results obtained 7 months following treatment revealed that GDNF alone was more efficacious than BDNF in protecting nigrostriatal neurons and improving behavioral deficits, and further, the combination of both factors did not provide more benefit than GDNF alone.

#### 2.5.2. Ischemia

During ischemic brain injury, or stroke, the lack of blood flow results in the deprivation of O_2_ and nutrients to neurons, glia, and other cell types within the brain. This initiates a cascade of events leading to the apoptotic demise of susceptible cells. Therefore, well-timed expression of anti-apoptotic genes and neuroprotective genes from HSV-1 amplicons under the regulation of HSV-1 viral promoters (e.g., IE 4/5 gene promoter) that initiate rapid, transient expression kinetics of the therapeutic gene affords a potential opportunity to protect neurons from post-ischemic death. Antonawich and colleagues demonstrated that unilateral hippocampal delivery of an HSV-1 amplicon over-expressing Bcl-2 (HSVbcl-2), an anti-apoptotic factor, in Mongolian gerbils 24 hours prior to bilateral common carotid artery occlusion resulted in the protection of CA1 hippocampal neurons compared to the control vector (HSVlac)-injected group [107]. Harvey and co-workers assessed the *in vivo* efficacy of the neurotrophic factor, GDNF, in protecting neurons in a focal ischemic stroke model [106]. Their study revealed that delivery of a GDNF over-expressing HSV-1 amplicon (HSVgdnf) into the cerebral cortex of rats 4 days prior to 60-min unilateral occlusion of the middle cerebral artery (MCOA) resulted in the reduction in ischemic tissue loss and the improvement of behavioral deficits compared to rats pre-treated with the control HSVlac amplicon. Delivery of the HSVgdnf amplicon 3-days post MCOA, however, did not serve to protect neurons from ischemia-induced death, thus highlighting GDNF’s neuroprotective role rather than neurorestorative activity in the setting of modeled ischemia. In addition to the above neuroprotective genes, antioxidant genes such as glutathione peroxidase gene (GPX) [108] and superoxide dismutase (SOD-1) [110], calbindin D28K gene [112], heat-shock protein gene HSP72 [109], and glucose transporter gene (GLUT) [111] have all been expressed via HSV-1 amplicons in animal models of stroke and have exhibited varied efficacy in protecting neurons following ischemic injury.

#### 2.5.3. Cancer therapy

A variety of anti-cancer therapies utilizing HSV-1 amplicon vectors are currently being investigated in preclinical animal models. Although conditionally replicating oncolytic HSV-1 vectors appear well suited for anti-cancer treatments due to their inherent ability to selectively replicate and lyse tumor cells, HSV-1 amplicons have also been utilized to deliver genes involved in anti-angiogenesis (e.g., soluble VEGF receptor (sFlk-1) [133], pro-drug activation (e.g., thymidine kinase) [117], apoptosis (e.g., TRAIL) [118], and immune enhancement (e.g., IL-2; [134] and IL-12; [135]). In addition, novel anti-cancer therapies using HSV-1 amplicons have been developed based on knowledge garnered with respect to molecular genetic changes that occur during tumorigenesis. For instance, alterations and over-expression of the epidermal growth factor receptor (EGFR) has been implicated in glial tumorigenesis [136]. Saydam and co-workers utilized the HSV-1 amplicon to express double-stranded hairpin RNA for targeted knockdown of EGFR in human Gli36 glioma cells [119]. Their study revealed that EGFR suppression inhibited tumor growth of proliferating Gli36 cells and also triggered apoptosis *in vitro* and *in vivo*. Recently, Tannous and co-workers used a HSV/EBV hybrid amplicon vector to express a constitutively open mutant, human brain sodium channel (ASIC2a) under the transcriptional control of the tetracycline regulatory system (Tet-on) [130]. Intratumoral delivery of this mutant sodium channel-expressing hybrid amplicon vector was shown to inhibit subcutaneous Gli36 tumor growth *in vivo*. This novel therapy involves the rapid killing of tumor cells upon doxycycline-mediated induction of ASIC2a, which causes an influx of Na^+^ ions and water leading to swelling and ultimately bursting of the tumor cell. The advantage of this therapy exists in its ability to rapidly kill a variety of tumor cells as well as a strong “bystander” killing effect.

Several groups have begun to utilize the “piggyback” HSV-1 amplicon/recombinant HSV-1 system, first introduced by Pechan and colleagues [137], as a means to provide enhancing functions to recombinant HSV-1 vectors to enable their replication and targeted killing of cancer cells. In a recent study, Lee and co-workers used an amplicon carrying a probasin-derived promoter (ARR(2)PB)-driven infected-cell polypeptide4 (ICP4) gene to complement viral replication and killing of prostate cancer cells by an ICP4-deficient herpes helper virus. Intratumoral delivery of this piggyback vector system in LNCap human prostate cancer xenografts resulted in 75% reduction in tumor volume and serum prostate specific antigen (PSA) [138].

#### 2.5.4. Hereditary ataxia

Recently, proof-of-concept gene replacement therapies utilizing HSV-1 amplicons have been reported for the treatment of inherited ataxias, including ataxia-telangiectasia (A-T) and Friedrich’s ataxia (FA). While mutations in the ataxia-telangiectasia mutated (ATM) gene give rise to this autosomal recessive disorder that is characterized by neurodegeneration, immunodeficiency and cancer predisposition [139], FA is primarily caused by a GAA triplet expansion within intron 1 of the FRDA gene, which ablates the expression of frataxin [140]. Loss of frataxin function causes significant neurodegeneration in the spinocerebellar system that is phenotypically characterized by reduced motor coordination. In a series of reports, Cortés and co-workers have demonstrated the ability of an HSV-1 amplicon harboring the 9-kb complementary DNA (cDNA) of ATM to correct specific aspects of the cellular phenotype in an A-T cell line [127], and to deliver the ATM cDNA into cerebellum-resident neurons of an ATM-deficient (Atm^−/−^) mouse [128]. The same group demonstrated stable integration of the ATM cDNA in the human AAVS1 locus of a transgenic Atm^−/−^ mouse harboring the human locus via a HSV/AAVrep^+^ hybrid amplicon vector [132]. However, the capacity for disease correction by replacing the ATM gene has not been possible in the Atm^−/−^ mouse model, since it does not recapitulate the neurodegenerative aspect of A-T seen in human patients.

Proof-of-concept studies for amplicon-based treatment of FA have been reported. Using a BAC-based HSV-1 amplicon carrying the entire FRDA locus (135-kb), Gomez-Sebastian and colleagues efficiently transduced FA patient primary fibroblasts and demonstrated functional recovery of these cells following exposure to oxidative stress [129]. Furthermore, Lim and co-workers generated a conditional frataxin transgenic mouse model using the Cre/*loxP* recombination system [125]. Expression from the “floxed” FRDA gene can be eliminated in a localized manner in the brainstem of this transgenic mouse via transduction of a Cre-expressing HSV-1 amplicon, which leads to a behavioral deficit in rotarod behavioral paradigm performance. Using this localized conditional gene knock-out model, Lim and colleagues demonstrated functional recovery of the associated behavioral deficits upon complementation of a human frataxin cDNA expressed via a HSV-1 amplicon. Collectively, these studies provide the foundation upon which HSV-1 amplicon-based therapeutic modalities can be devised for the treatment of A-T and FA, which are currently refractory to available therapies.

### 2.6. Use of HSV-1 amplicons for vaccine development

The HSV-1 amplicon has been vetted in recent years as a potential vaccine platform for cancer, human immunodeficiency virus (HIV) and Alzheimer’s disease (AD). Immunotherapeutics designed to specifically target pathogens that underlie currently intractable, chronic diseases have shown promise in preclinical testing and clinical trials. Helper virus-free HSV-1 amplicons are ideally suited to deliver such antigens, since they inherently elicit minimal anti-vector immune responses within the host, exhibit cellular tropism for professional antigen-presenting cells (APCs), package and deliver large DNA segments, and transiently express immunogenic factors to acutely prime and shape the host’s immune system.

#### 2.6.1. Cancer vaccines

A wide spectrum of cancers ranging from B-cell lymphoma to prostate cancer has been targeted for HSV-1 amplicon-mediated immunotherapy. Early strategies involved the delivery of potent cytokines via HSV-1 derived amplicons directly into the tumor environment to elaborate a localized enhancement of APCs for tumor antigen presentation and CTL activation for targeted clearance of tumor cells. Toda and colleagues used a murine granulocyte-macrophage colony-stimulating factor (GM-CSF)-encoding HSV-1 amplicon packaged using a mutant helper virus (*tsK*) to treat a bilateral subcutaneous tumor model of Harding-Passey murine melanoma [124]. They demonstrated that secretion of GM-CSF from the transduced tumor cells resulted in a vector dose-dependent retardation of tumor growth in the amplicon-injected tumor as well as the contralateral uninjected tumor, and concomitant extension of mouse survival rate. In a separate study, Herrlinger *et al.* utilized a GM-CSF-expressing, helper virus-free HSV-1 amplicon (HSVGM) in combination with polybrene to transduce glioblastoma cells (GL261), which were subcutaneously injected into C57BL/6 mice that 7 days later were implanted with wild-type GL261 glioma cells [120]. This prophylactic vaccination paradigm resulted in 60% long-term survivors (>80-days) compared to 10% long-term survivors in the groups vaccinated with equivalent number of non-transduced GL261 cells or ”empty-vector”-transduced GL261 cells. Tolba and co-workers further extended cytokine-based HSV-1 amplicon vaccination therapy by delivering another potent chemokine, secondary lymphoid tissue chemokine (SLC), alone, or in combination with a co-stimulatory ligand (e.g. CD40L) via HSV-1 amplicons in murine models of B cell lymphoma (A20) and adenocarcinoma (CT-26) to enhance tumor-specific APC recruitment and maturation in lymph nodes for the elicitation of a T-cell-mediated anti-tumor responses [123]. Co-delivery of the HSV-SLC and HSV-CD40L amplicons significantly decreased tumor volume and increased survival rates in both tumor models.

Another amplicon-based approach that has been used to trigger a cytotoxic T-lymphocyte (CTL) mediated anti-tumor immune response has been to modify dendritic cells *ex vivo* via transduction of HSV-1 amplicons expressing specific tumor antigens. These vector-transduced dendritic cells once transplanted in the host can act as potent antigen-presenting cells and are capable of inducing a CTL response that would specifically target tumor antigen-presenting cancer cells for destruction. This strategy was adopted for the treatment of a murine model of prostate cancer wherein a helper virus–free HSV-1 amplicon expressing the prostate-specific antigen (HSV-PSA), which is a tumor antigen specifically expressed by prostate epithelial and carcinoma cells, was shown to be efficient in transducing dendritic cells (DCs) and processing the tumor antigen for MHC class II presentation to CTL [121]. Subsequent immunization of mice with the HSV-PSA-transduced DCs resulted in the induction of a specific CTL response, and also protected these mice from subsequent challenge with tumors expressing PSA.

#### 2.6.2. HIV vaccines

HSV-1 amplicons have also been useful in devising vaccine delivery platforms for human immunodeficiency virus (HIV-1). The principle for such an endeavor came about from the work of Hocknell and co-workers, who demonstrated that intramuscular delivery of a helper virus-free HSV-1 amplicon encoding the HIV-1 envelope glycoprotein 120 (HSV:gp120) elicited a robust gp120-specific CD8+ T-lymphocyte and humoral response in mice, which persisted at 171-days following a single inoculation [141]. They also showed that prior exposure to a wild-type HSV-1 strain did not hinder the generation of a envelope-specific cellular immune response, but did somewhat impair anti-GP120 humoral responses in mice following vaccination with the HSV:gp120 vector, thereby addressing the concern regarding the efficacy of this vaccine platform in the presence of pre-existing anti-HSV-1 neutralizing antibodies. In a recent study, Gorantla and colleagues used a nonobese-diabetic severe combined immunodeficiency syndrome mouse model that had been repopulated with human peripheral blood lymphocytes (hu-PBL-NOD/SCID) to test the efficacy of a vaccination paradigm using autologous dendritic cells transduced *ex vivo* with a helper virus-free HSVgp120_MN/LAI_ amplicon vector [113]. *In vitro* transduction of human DCs with the HSVgp120 _MN/LAI_ amplicon resulted in partial maturation of the DCs with increased expression of the major histocompatibilty complex II (HLA-DR), co-stimulatory molecules (CD80 and CD86), and the maturation antigen (CD83) compared to nontransduced DC controls as revealed by flow cytometric analysis. Subsequent engraftment of the HSVgp120 _MN/LAI_-transduced DCs in hu-PBL-NOD/SCID mice resulted in a gp120-specific cellular and humoral response that peaked at day 7 following immunization. Furthermore, an infectious HIV challenge performed on these immunized mice at their peak immune response time (*i.e.* on day 7 following immunization) with either a HIV-1_ADA_ strain or a HIV-1_LAI_ strain resulted in partial protection against only the HIV-1_ADA_ strain as determined by neutralizing antibody titers and protection of CD4^+^ T cells. This work also validated the use of the mouse-human chimeric hu-PBL-NOD/SCID model to evaluate immune responses to DC-mediated vaccination paradigms to regulate HIV infection.

#### 2.6.3. Alzheimer’s disease vaccines

Alzheimer’s disease is a progressive neurodegenerative disease, and given the recent findings pertaining to its etiology represents another potential candidate for HSV-1 amplicon-based immunotherapy. Recent findings have established that the amyloid-β 1–42 amino acid peptide species (designated Aβ_1–42_), which is an aberrant cleavage product of the amyloid-precursor protein (APP), has significant implications in the etiology of the disease [142]. Therefore, immunotherapeutic strategies designed to prevent Aβ accumulation and/or its subsequent fibrillization have been shown to be viable in retarding the progression of the disease in preclinical AD mouse models [143–146]. The initial development of an active Aβ_1–42_-directed vaccine based on a peptide and adjuvant vaccination platform revealed promising preclinical results in animal models of AD. However, a subsequent Phase II clinical trial using the AN-1792 Aβ peptide/molecular adjuvant-based vaccine elicited a vigorous inflammatory response in a subset of patients [147,148]. This highlighted the need to develop alternate vaccination platforms that could not only break self-tolerance to the Aβ self-peptide, but also deliver immunomodulatory molecules that could “shape” the host’s immune response down a Th2 path to obviate such deleterious inflammatory responses. The initial generation and peripheral administration of HSV-1 amplicons designed to express Aβ_1–42_ alone (HSVAβ) or Aβ_1–42_ fused to the molecular adjuvant tetanus toxin Fragment C (HSVAβ/TtxFC) in the Tg2576 AD mouse model revealed an enhancement in humoral responses to Aβ and reduced Aβ deposition in the brains of these mice [115]. However, the HSVAβ amplicon also elicited an increase in the transcript levels of specific pro-inflammatory molecules (e.g. IFN-β, IFN-γ, IL-6, and MIP-2) within the hippocampi of these mice, which highlighted the purported need to bias the immune system toward a Th2 response pathway. In a recent follow-up study, Aβ_1–42_ was co-delivered with interleukin-4 (IL-4), a cytokine competent in promoting a Th2-biased immune response, via a helper virus-free HSV-1 amplicon (designated HSV_IE_Aβ_CMV_IL-4) into the triple-transgenic mouse model of AD (3xTg-AD), a model that closely recapitulates the pathological hallmarks observed in human AD [116]. Vaccination of 3xTg-AD mice at 2, 3, and 9 months of age via subcutaneous administration of the HSV_IE_Aβ_CMV_IL-4 amplicon or the control amplicons, HSV_IE_Aβ_CMV_2 and HSV_IE_1_CMV_2 revealed that HSV_IE_Aβ_CMV_IL-4 vaccinated mice exhibited an increased level of Aβ_1–42_-specific IgG1 isotype antibodies indicative of a Th2 response, significantly suppressed Aβ deposition and tau hyperphosphorylation within the hippocampus, and performed significantly better than the control groups in the Barnes Maze spatial learning and memory paradigm at the 11-month time-point drawing a strong correlation between reduced AD-related pathology and improved behavioral performance. These promising results provide ample impetus for further research into HSV-1 amplicon-based vaccine development for the treatment of AD.

### 2.7. Current impediments to clinical implementation of HSV-1 amplicons

#### 2.7.1. Scale-up challenges

One of the major challenges facing HSV-1 amplicon-mediated gene therapy in the clinical arena is the inability to produce high-titer amplicon vector stocks with currently employed packaging technologies. As discussed previously, helper virus-based methods have undergone significant improvements and are currently capable of producing amplicon titer at ∼10^9^ TU/ml after concentration albeit with trace amounts of helper virus contamination. Since, even minute numbers of helper virus particles pose a risk in a clinical setting, further improvements are required. The use of multiply deleted helper viruses or enhancements to the Cre/*loxP*-disabled helper virus strategy may provide a means to generate safer amplicon stocks. Helper virus-free BAC- and cosmid-based packaging methods produce “clean” amplicon stocks free of contaminating helper virus, but this is achieved at a price of even lower titers ranging from 10^7^–10^8^ TU/ml after concentration. The inability to generate high-titer helper virus-free amplicon stocks stems from the inefficiencies associated with current lipid-based transfection methods used to deliver large DNA molecules such as the HSV-1 genome-encoding BAC (150 to 175-kb) into standard packaging cell lines. Furthermore, current FDA-approved standard mammalian cell lines used for amplicon packaging (e.g. Vero cells) may not be optimal for generating high-titer HSV-1 amplicon stocks. Thus, more efficient methods of delivering the BAC into mammalian cells along with more “packaging-optimized” mammalian cell lines need to be employed if high titer, helper virus-free amplicon production is to become a reality.

#### 2.7.2. Extension of gene expression duration

As mentioned previously, the duration of transgene expression achieved from the conventional HSV-1 amplicon varies, due to episomal maintenance and host-cell induced silencing of the amplicon-encoded transgene. Furthermore, it has been shown that helper virus-generated amplicons support relatively longer term transgene expression compared to helper virus-free amplicon stocks possibly due to the presence of viral gene products expressed by the contaminating helper virus (e.g. ICP0). Thus, conventional HSV-1 amplicons are limited in their utility as gene replacement vectors in a clinical setting for diseases that require the therapeutic gene to be expressed over a patient’s life span. Viral promoters, such as the HSV-1 IE 4/5 promoter and the cytomegalovirus (CMV) immediate-early enhancer/promoter, have been shown to be transcriptionally repressed within 1–2 weeks post-transduction in neurons. In contrast, certain neuronal subtype-specific cellular promoters such as those derived from preproenkaphalin (ENK) [149], rat tyrosine hydrolase (TH) [150,151], glutamic acid decarboxylase 67 (GAD 67) [152], phosphate-activated glutaminase (PAG) [152], vesicular glutamate transporter-1 (VGLUT1) [152], and the chimeric TH-NFH [83,104,105,153,154] have shown promise in extending transgene expression ranging from 2–14 months in specific neuronal populations in the brain. Furthermore, the incorporation of mammalian insulator elements such as the well-characterized chicken-β-globin insulator into HSV-1 vectors has stabilized long-term expression from the TH-NFH promoter as demonstrated by Zhang and colleagues [153]. Thus, the potential exists to generate optimized forms of promoter and chromatin remodeling elements that support long-term and cell type-specific transgene expression that would be of significant benefit in a clinical setting for chronic disorders. Besides the use of cellular promoters to support long-term transgene expression, the development of integration-competent hybrid amplicon vector platforms has sparked enthusiasm in the field to achieve stable long-term transgene expression. However, testing of these platforms is still in its infancy. Further development and fine-tuning of these newer HSV-1 amplicon-based vectors is required to ensure that these hybrid platforms maintain host cell genomic integrity by integrating the transgene into “safe” chromosomal locations and also ensure that long-term expression of a given therapeutic gene would not lead to deleterious side effects.

#### 2.7.3. Regulated transgene expression

With the development of HSV-1 amplicon vectors that mediate long-term transgene expression comes the need for finely tuned transgene regulation, since chronic expression of a given therapeutic transgene may prove to be deleterious and an “exit strategy” may be warranted. Several inducible expression systems have been successfully adapted for use in the HSV-1 amplicon. Since the initial testing of a glucocorticoid-inducible system to regulate transgene expression from a HSV-1 amplicon by Lu and colleagues [155], investigators have employed the tetracycline-responsive promoter system in the setting of an amplicon to obtain stringent control over transgene expression [130,156,157]. Furthermore, the extensive transgene capacity of the HSV-1 amplicon has allowed for the adaptation of the rapamycin-based “dimerizer” system into a single amplicon vector for transgene regulation [158]. While these regulatory systems appear to provide a means to efficiently induce/repress expression of the therapeutic gene when required, further *in vivo* testing is warranted to ensure that stringent transgene expression control is maintained during “on” and “off” states pertaining to each inducer/transgene system and that the various components of the regulatory system do not lead to elicitation of untoward host immune/inflammatory responses.

## 3. Conclusions

The HSV-1 derived amplicon vector has generated a special niche for itself in the field of gene therapy due to its extensive transgene capacity, broad-range cellular tropism, reduced immunogenicity, and ease of manipulation. From its simplistic design, which demands only two cis-acting non-structural elements from the wild-type HSV-1 genome to its ability to harbor and deliver entire genomic loci for gene replacement purposes has propelled its utility in gene transfer paradigms. Moreover, the derivation of improved helper virus-free packaging strategies has accelerated preclinical testing of amplicons in a number of animal models of human disease, but significant efforts must be invested to create and logistically test scalable procedures for future clinical implementation of this vector platform. While the field of gene therapy has made a number of significant advances during the past decade, several difficult lessons have been learned along the tenuous path to the clinic. These lessons in caution have periodically re-focused the field to address critical issues requisite for the successful and safe implementation of current and future viral vector-based therapeutic interventions. To this end, the preclinical promise of the HSV-1 amplicon warrants a methodical and rigorous approach for assessing the platform’s limitations, exploiting its strengths, and optimizing its *in vivo* safety profile as it moves forward toward eventual clinical testing.

## Figures and Tables

**Table 1.** Types of HSV-1 derived amplicon vector platforms that have been preclinically tested.

**Amplicon**	**Type**	**Episomal /Integration-Competent**	**Disease Application**	Transgene(s) Delivered (** indicates delivery of entire genomic locus)	**References**

**HSV-1**	Conventional	Episomal	Parkinson’s disease	GDNF	[103]
				BDNF	[103]
				AADC	[104]
				TH	[104]
				GTP CH1	[105]
				VMAT -2	[105]

			Ischemia	GDNF	[106]
				bcl-2	[107]
				GPX	[108]
				HSP72	[109]
				SOD-1	[110]
				GLUT1	[111]
				Calbindin d28k	[112]

			HIV	gp120	[113]
					[114]

			Alzheimer’s disease	Amyloid-β (1-42)	[115]
				IL-4	[116]

			Glioblastoma	Thymidine kinase	[117]
				TRAIL	[118]
				siRNA α -EGFR	[119]
				GM-CSF	[120]

			Prostate cancer	Prostate-specific antigen (PSA)	[121]

			Chronic Lymphocytic Leukemia	CD80 (B7.1)	
				CD154 (CD40L)	[122]
				SLC	[123]
				CD40L	

			Melanoma	GM-CSF	[124]

			Friedreich’s Ataxia	FRDA cDNA	[125]

			Ataxia Telangiectasia	ATM cDNA	[126] [127,128]

**HSV/EBV**	Hybrid	Episomal	Friedreich’s Ataxia	FRDA**	[129]

			Glioblastoma	ASIC2α	[130]

			Lesch-Nyhan syndrome	HPRT**	[131]

			Familial hypercholesterolemia	LDLR**	[49]

**HSV-BAC-S/MAR**	Hybrid	Episomal	Familial hypercholesterolemia	LDLR**	[50]

**HSV HAC**	Hybrid	Episomal	Lesch-Nyhan syndrome	HPRT**	[51]

**HSV/EBV/RV**	Tribrid	Episomal	Glioblastoma	RV gag-pol, env genes	[60]
					[62]

**HSV/AAV/RV**	Tribrid	Integration-competent	Glioblastoma	RV gag-pol, env genes	[61]

**HSV/AAV**	Hybrid	Integration-competent	Ataxia Telangiectasia	ATM cDNA	[132]

			Glioblastoma	β-galactosidase	[53]

			Lysosomal storage disease	Human β-alactosidase**	[57]

**HSV/Sleeping Beauty**	Hybrid	Integration-competent	*In utero* delivery	β-galactosidase-neomycin phosphotransferase	[63]
					[64]
